# Current Insights on Salivary Gland Adenoid Cystic Carcinoma: Related Genes and Molecular Pathways

**DOI:** 10.3390/genes16040370

**Published:** 2025-03-24

**Authors:** Vasileios Zisis, Konstantinos Poulopoulos, Nikolaos Shinas, Christina Charisi, Athanasios Poulopoulos

**Affiliations:** 1 Department of Oral Medicine and Pathology, Dental School, Aristotle University of Thessaloniki, 541 24 Thessaloniki, Greeceakpoul@dent.auth.gr (A.P.); 2 Department of Oral and Maxillofacial Radiology, Henry M. Goldman School of Dental Medicine, Boston University, Boston, MA 02215, USA

**Keywords:** salivary gland, salivary tumor, salivary cancer, adenoid cystic carcinoma, adenocystic carcinoma, genes, genetic, molecular pathway, oral cancer, head and neck cancer

## Abstract

**Background/Objectives**: Salivary adenoid cystic carcinoma (ACC) is a rare but aggressive neoplasm that predominantly arises from the salivary glands, accounting for a significant proportion of salivary gland cancers. The aim of this literature review is to illustrate the current insights on ACC with regards to related genes and molecular pathways by analyzing original research articles from the period 2015–2025. **Methods**: An electronic search of literature was performed between January and February 2025 to identify all articles investigating the current insights on salivary gland adenoid cystic carcinoma and its related genes and molecular pathways. The search was conducted using MEDLINE (National Library of Medicine)-PubMed with restrictions concerning the date of publication. In particular, we focused on the period 2015–2025 using the following keywords: Salivary gland adenoid cystic carcinoma AND genes AND molecular pathways. This was followed by a manual search, and references were used to identify relevant articles. **Results**: In total, 41 articles were identified through the keywords. After the implementation of the time frame 2015–2025, 31 articles remained. Subsequently, by reading the titles and abstracts and thereby excluding non-original research articles and articles written in a language other than English, 23 articles remained. **Conclusions**: These studies identified 23 relevant genes or pathways whose analysis yielded the most recent data regarding their function. The classification of ACC is multifaceted, encompassing distinct histological subtypes that are crucial for determining prognosis and treatment approaches. Current oncological practices classify ACC based on these histological features alongside emerging genetic and molecular markers that promise to enhance our understanding of the disease’s biology. Diagnostic strategies have evolved, leveraging techniques such as biopsy and molecular diagnostics, which have significantly improved the detection and characterization of ACC. Regarding treatment, the management of ACC remains a challenge due to its propensity for local invasion and metastasis, with surgery, radiation, and chemotherapy being the mainstays of therapy. The development of targeted therapies based on ACC’s molecular profile will allow for a better prognosis and an enhanced quality of life of patients.

## 1. Introduction

Salivary adenoid cystic carcinoma (ACC) is a rare but aggressive neoplasm that predominantly arises from the salivary glands, accounting for a significant proportion of salivary gland cancers. The incidence and prevalence rates of ACC demonstrate significant variability across different geographic regions. In western Europe, ACC is noted as the most prevalent salivary gland malignancy [1]. Conversely, in the United States, ACC accounts for less than 1% of head and neck neoplasms and ranks third among salivary gland malignancies, following mucoepidermoid carcinoma and polymorphous adenocarcinoma [1]. A declining incidence of ACC in the United States has been observed over recent decades, which may reflect advances in diagnostic techniques [1].

Gene fusions, such as MYB–NFIB, MYBL1–NFIB, and MYBL1–RAD51, play a crucial role in the development of ACC [1]. Additionally, intraoral minor salivary gland ACC may have a more favorable prognosis compared to major gland ACC and ACC of the sinonasal tract [1]. ACC manifests a distinct female predilection [1].

The major histological subtypes of ACC encompass a spectrum of patterns: the tubular, cribriform, and solid patterns are key subtypes, with the solid pattern being associated with a more aggressive clinical course and a worse prognosis [2,3]. The presence of solid areas within the tumor is a crucial factor in the grading of ACC, as it correlates with increased malignancy and a heightened risk of metastasis [3]. The cribriform pattern, recognized as the second major subtype, contributes to the unique histological diversity of ACC and is characterized by its sieve-like architecture [4]. The cribriform variant of ACC is distinguished by its oval-to-rounded masses of basaloid cells interspersed with microcystic spaces filled with pink or bluish material [5]. The solid variant is characterized by sheets of basaloid cells lacking lumina, contrasting with the tubular variant that presents ducts and tubules lined with luminal cells and surrounded by non-luminal myoepithelial cells [5].

ACC is characterized by a prolonged yet relentlessly progressive clinical course, which often leads to delayed diagnosis and treatment [6]. This slow progression can result in the tumor infiltrating surrounding tissues, thus causing symptoms such as persistent pain or swelling in the affected area. ACC rarely presents with regional lymph node metastasis, which can mislead clinical evaluations and impact treatment decisions [6]. High-grade tumors tend to have a more aggressive clinical course and poorer prognosis, underscoring the importance of histological evaluation in predicting patient outcomes [6].

Magnetic resonance imaging (MRI) plays a significant role in the initial detection and evaluation of ACC, providing detailed visualization of the tumor and its extent, which is crucial for planning treatment strategies [7]. Histopathological examination is another essential component, where the presence of cribriform or multicystic areas is frequently observed, distinguishing ACC from other types of adenocarcinomas [8]. Immunohistochemical (IHC) analysis adds another layer of specificity, with markers such as nuclear MYB overexpression offering diagnostic usefulness, although variability in staining can pose challenges [9].

The treatment of ACC is multifaceted, with surgical resection being the cornerstone of management. Radical surgical resection, aimed at achieving tumor-free margins, is considered the “gold standard” for ACC of the head and neck [10]. However, due to the high propensity for local recurrence and perineural invasion typical of ACC, adjuvant radiotherapy (RT) is frequently recommended to bolster locoregional control after surgery [11,12]. This approach is particularly beneficial in decreasing the risk of difficult-to-salvage failures at the base of the skull, a common site for recurrence given the tumor’s invasive nature [12]. In cases where the disease is deemed unresectable, definitive radiotherapy becomes the primary treatment modality, necessitating high radiation doses to achieve tumor control, albeit at the risk of late-onset local toxicities [12]. While the incorporation of adjuvant chemotherapy into ACC treatment regimens is debated, with guidelines generally advising against routine use outside of clinical trials, it may be considered for high-risk patients and is actively being investigated in ongoing studies [11]. Targeted molecular therapies might offer additional avenues for improving outcomes in ACC management [13]. Overall, a multidisciplinary approach considering disease burden, potential relapses, and treatment-related toxicities is crucial in the management of ACC, emphasizing the need for tailored treatment plans and continued exploration of innovative therapies [12].

The aim of this literature review is to illustrate the current insights on ACC with regard to related genes and molecular pathways by analyzing original research articles from the period 2015–2025.

## 2. Materials and Methods

An electronic search of literature was performed between January 2025 and February 2025 to identify all articles investigating current insights on salivary adenocystic carcinoma and its related genes and molecular pathways. The search was conducted using MEDLINE (National Library of Medicine)-PubMed with restrictions concerning the date of publication. In particular, we focused on the period 2015–2025 using the following keywords: Salivary gland adenoid cystic carcinoma AND genes AND molecular pathways. This was followed by a manual search, and references were used to identify relevant articles. The articles identified from the electronic and manual search were screened to eliminate those that failed to meet the respective inclusion and exclusion criteria listed below.

### Inclusion and Exclusion Criteria

The electronic search was based on the following inclusion criteria:

1. Language: Articles written in English.
2. Time frame: Studies published between 2015–2025
3. Type of study: Original research articles

The following exclusion criteria were applied:

1. Language: Articles written in a language other than English
2. Time frame: Articles written before 2015
3. Type of study: Non-original research articles

The authors VZ, KP, NS, CC screened and assessed the articles independently and reached the same conclusions.


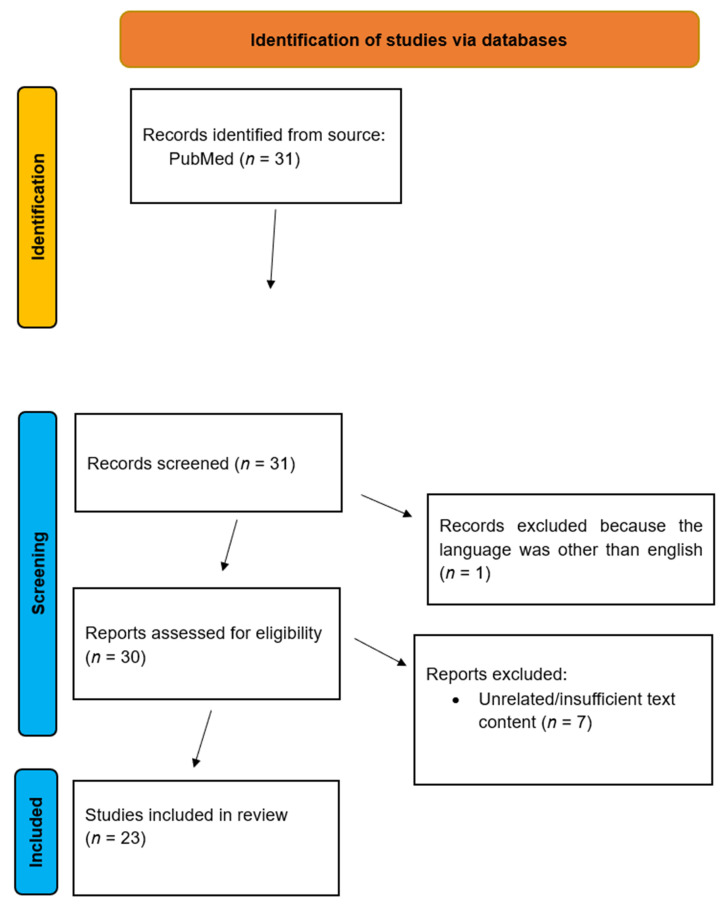


In total, 41 articles were identified through the keywords. After the implementation of the time frame 2015–2025, 31 articles remained. Subsequently, by reading the titles and abstracts, and thereby excluding non-original research articles and articles written in a language other than English, 23 articles remained (Table 1).

## 3. Results

The features of the included articles are summarized in Table 2.

The key findings of our review are the following: Protein/pathway alterations may lead to different subtypes (ACC-I and ACC-II) and prognosis. Epigenetic changes in ACC (NFIB–MYB rearrangement and NOTCH mutation) correlate with worse prognosis. Differential expressions of genes, miRNAs and heterogeneity also play a role in the emergence of ACC. Genetic aberrations, translocations and fusions may be observed in the majority of cases. The role of NOTCH is critical: inhibiting the NOTCH1–HEY1 pathway has therapeutic value and HES1, a gene of NOTCH1, contributes to ACC development. RAS mutations are common in ACC and associated with a poor prognosis for the patient. MYB TSS2 activity is associated with metastases. TGFβ genes contribute to the process of tumorigenesis.

Regarding therapy, retinoic acid and triptonide may constitute a part of the therapeutic regimen against ACC. Regulating Wnt (f.e. through triptonide administration) and silencing the PI3K pathway serve as therapeutic targets. PP2A inhibition leads to ACC due to mTOR signaling activation. Targeting the alternative MYB promoter prevents ACC development. Suppression of RAR/RXR signaling constitutes a possible therapeutic target. The synergy of PTEN and SMAD4 necessitates targeting mTOR and/or TGFβ signaling. FGFR1 variants function through the AXL/AKT signaling pathway independent of FGF/FGFR1, thus combining FGFR1 and AXL inhibition as an effective ACC therapy.

## 4. Discussion

The molecular subtyping of ACC shows distinct heterogeneity. Two primary molecular subtypes are distinguished by the expression of MYB and MYBL1 oncogenes [37]. These subtypes exhibit overlapping gene expression, indicating a shared oncogenic pathway through the targeting of SOX4 and EN1 genes by Myb proteins [37]. In 20% of ACC cases a distinct gene expression signature akin to embryonic stem cells presents, correlating with poor overall survival of less than 30 months [37]. This finding underscores the importance of gene expression profiling in ACC for identifying high-risk patients who may benefit from targeted therapies [38].

In solid-type ACC, there is an inactivation of several critical signaling pathways [39]. This suggests a distinct molecular landscape, thus necessitating a different therapeutic approach compared to cribriform-tubular or cribriform types [39].

The TGF-β signaling pathway is integral to the development and progression of salivary gland tumors [40]. At the core of this process is the epithelial-mesenchymal transition (EMT), which is a hallmark of fibrosis and is largely driven by TGF-β as the master regulator [40]. The activation of SMAD-mediated pathways facilitates EMT in response to TGF-β1, contributing to the fibrotic process within salivary glands [40]. While SMAD pathways are vital, non-SMAD signaling pathways also contribute to the cellular responses elicited by TGF-β [40]. Increased TGF-β signaling is linked to duct ligation [41]. The alteration in the TGFβ signaling pathway shifts its function from tumor-suppressive to pro-oncogenic [42]. This switch is accompanied by changes in the PI3K/AKT pathway and the inhibition of mTOR, which collectively contribute to the minimization of microvascular density and suppression of tumor growth [43]. Targeting the TGFβ signaling pathway mitigates radiation-induced damage to the glands. Radiotherapy negatively impacts salivary gland function, leading to debilitating side effects such as xerostomia or dry mouth [44]. By intervening in the TGFβ signaling pathway, it may be possible to protect the salivary glands [44]. Simultaneously modulating Wnt signaling could further inhibit tumor growth or progression, providing a synergistic approach for the treatment of salivary gland tumors [44].

MYB–NFIB gene fusion enhances MYB transcriptional regulatory activity, thus promoting tumorigenesis [45]. In addition to MYB–NFIB fusion, mutations in the SPEN gene, which encodes an RNA-binding coregulatory protein, involve the NOTCH and FGFR pathways [45]. Therefore, ACC may be driven both by the well-documented MYB and NFIB gene alterations and by mutations in various chromatin regulatory genes [45]. These mutations may involve genes not previously linked to cancer, highlighting unique oncogenic pathways in ACC [45]. A considerable subset of ACCs lacks the MYB-NFIB translocation, suggesting that other genetic factors may drive the disease’s progression [46]. Powell et al. revealed that such epigenetic changes in ACC correlate with worse prognosis [16]. The interplay of MYB with mutations in the FGF–IGF–PI3K pathway observed in 30% of ACC tumors reveals a complicated molecular background [47]. The mutations in chromatin-state regulators show the importance of epigenetic changes in ACC [47].

Key hub genes such as SLC22A3, FOXP2, Cdc42EP3, COL27A1, DUSP1, and HSPB8 are central players in ACC due to their involvement in signaling pathways correlated to ACC, such as SOX2, AR, SMAD, and MAPK [27]. HES1 contributes to ACC’s aggressiveness and poor prognosis [48]. HES1 is linked to the Notch signaling pathway, which is crucial for maintaining cell proliferation and differentiation in salivary glands [49]. HES1, HES2 and HEY2 enhance the cell proliferation rate, as evidenced by an increase in the Ki-67 index [50]. The interplay between HES1 and MYC has been identified as a pivotal factor in promoting tumorigenesis [51]. The NOTCH1–HEY1 pathway guides the processes of self-renewal and epithelial-mesenchymal transition (EMT) [52,53]. EMT is a cellular program that is often hijacked by cancer cells to enhance their invasiveness and metastatic potential [54].

EGFR gene mutations associated with tumorigenesis are absent in ACC, even when a metastasis has been detected [55]. The absence of mutations suggests that EGFR may not play a direct oncogenic role in ACC. RAS gene mutations were similarly significant contributors to the pathogenesis of ACC [56]. The activation of the KRAS signaling pathway has been linked to aggressive tumor behavior and poor prognosis in ACC patients [57]. The significance of PIK3CA, BRAF, and AKT1 gene mutations influences the PI3K/AKT/mTOR and MAPK signaling pathways, which are pivotal in tumorigenesis. The PIK3CA mutation is prevalent in salivary gland tumors [58]. The co-occurrence of PIK3CA mutations with HRAS mutations suggests a parallel activation of the PI3K and MAPK pathways, which reinforces the need for targeted parallel therapies [58]. The identification of BRAF V600E opens the possibility of utilizing BRAF inhibitors for targeted therapy [58].

FGFR1 splice variants modulate signaling pathways that promote cellular proliferation and survival [59]. These variants may be coexpressed, enhancing FGF signaling [59,60]. The regulation of splicing influences cancer progression [61]. FGFR1-IIIc is implicated in aggressive behavior in cancers such as prostate cancer [62]. FGFR1 variants cooperate with AXL and desensitize cells to the FGFR1 inhibitor, suggesting parallel targeted therapy [29]. PP2A functions as a tumor suppressor in salivary gland malignancies. PP2A is a serine/threonine phosphatase that plays a critical role in regulating cell cycle and signal transduction pathways, such as the PI3K pathway, leading to cancer when dysregulated [63]. In ACC, PP2A’s tumor-suppressive ability is often compromised, either through genetic mutations or by inhibition through external factors [64], thus enhancing ACC’s malignant potential [65]. Subunit B55a of PP2A is particularly significant in this process [66].

Detecting fusion genes in salivary gland cancer (SC) patients involves RNA-based next-generation sequencing (NGS) [67]. Additionally, the FusionPlex Solid Tumor kit (ArcherDX), another NGS tool, has been utilized to analyze cases of SC, demonstrating its effectiveness in detecting fusion genes across a broad spectrum of genetic variations [68]. Fluorescence in situ hybridization (FISH) is frequently employed to investigate specific chromosomal regions for abnormalities associated with gene fusions [69].

The microRNA expression patterns reveal critical insights into the underlying molecular mechanisms of ACC. The downregulation of miR-181a-5p, which leads to lymph node metastasis, plays a pivotal role, thereby constituting a viable therapeutic target [70]. MiR-181a-5p is linked with the upregulation of hsa_circRNA_001982, suggesting a complex interaction network that might influence tumor progression [70]. The expression of the MYB or MYBL1 genes was found to be intricately linked with the expression of SOX4 and EN1, suggesting that these genes are direct targets of Myb proteins [37]. The increased expression of genes such as EN1, FABP7, MYB, and VCAN in ACC as compared to normal tissues further underscores the role of these mRNA expression profiles [71].

Proteogenomic analysis distinguishes between subtypes by integrating genomic data from exome sequencing with proteomic data derived from mass spectrometry, allowing for a comprehensive view of protein expression and mutation profiles [72]. This method involves isolating exosomes and ectosomes from SH-SY5Y cells and performing label-free quantitative proteomics, which helps in examining the differential expression of proteins across these subtypes [72].

Retinoic acid (RA) signaling plays a multifaceted role in lung metastasis. The ability of RA to modulate a switch between cellular proliferation and differentiation highlights its pivotal role in metastatic progression. It has been observed that the influence of RA on key signaling pathways is more pronounced in primary tumor cells compared to metastatic cells, suggesting a differential impact based on the stage of cancer progression [73]. Furthermore, RA signaling’s involvement in epithelial differentiation, particularly through the TGF-β/CTGF pathway, underscores its critical function in maintaining epithelial integrity and potentially inhibiting metastasis in lung cancer [74].

## 5. Conclusions

ACC constitutes a rare and aggressive neoplasm that predominantly arises from the salivary glands. Its unique clinical characteristics, often presenting with insidious symptoms, make early diagnosis challenging and contribute to its complex epidemiological profile. The classification of ACC is multifaceted, encompassing distinct histological subtypes that are crucial for determining prognosis and treatment approaches. Current oncological practices classify ACC based on these histological features, alongside emerging genetic and molecular markers that promise to enhance our understanding of the disease’s biology. Diagnostic strategies have evolved, leveraging techniques such as biopsy and molecular diagnostics, which have significantly improved the detection and characterization of ACC. Regarding treatment, the management of ACC remains a challenge due to its propensity for local invasion and metastasis, with surgery, radiation, and chemotherapy being the mainstays of therapy. The development of targeted therapies based on ACC’s molecular profile will allow for a better prognosis and an enhanced quality of life of patients.

## Figures and Tables

**Table 1:** Studies included in the comprehensive review.

41 articles through keywords	
Time frame 2015–2025	
31 articles remained	10 articles excluded
The rest of the inclusion criteria	
23 articles remained	8 articles excluded

**Table 2:** Summary of the main findings and characteristics of the original studies included in our comprehensive review.

Study	Country	Sample Size	Method	Results
[14]	USA	54/38	RNA/DNA sequencing (54)/ RPPA (38)	ACC-I (37%) and ACC-II (63%). ACC-I: worse prognosis. ACC-II: better prognosis.
[15]	China	79	scRNA-seq and exome target capture sequencing	ATRA suppresses lung metastasis due to aberrant NOTCH1 or MYB expression.
[16]	Ireland	11	In situ hybridization, DNA sequencing, and bioinformatic analysis	Three-quarters of the tumors displayed NFIB-MYB rearrangement at the 6q23.3 locus; one-third displayed NOTCH mutations.
[17]	China	15	Gene set enrichment analysis	Identified 382 DEGs: 119 upregulated and 263 downregulated genes.
[18]	USA	34	Whole-genome sequencing and variant analyses	Mutations in NOTCH/SPEN and t(6;9) associated gene fusions.
[19]	China	87	IHC, PCR, Cell assay, xenograft tumor model.	NOTCH1–HEY1 pathway upregulated in ACC and increased expression of EMT-related genes and MMPs.
[20]	Sweden	26	RNA sequencing and PCR	MYB TSS2 activity higher in ACC metastases (*p* = 0.0003).
[21]	China	60	RNA sequencing and IHC	HES1 linked to NOTCH. Silencing HES1 expression leads to suppression of cell metastasis and invasion.
[22]	China	3	Pathway enrichment analysis	Identified 27 upregulated and 54 downregulated mRNAs.
[23]	Netherlands	46	NGS	Aberrations in PIK3CA (*n* = 18, 15%), ERBB2 (*n* = 15, 12%), HRAS and NOTCH1. Fusion transcripts were detected in 50% of the cases.
[24]	China	6	NGS, IHC, PCR, and bioinformatics	SCUBE3, CA6, hsa-miR-885-5p play an essential role in the development of ACC.
[25]	China	-	Molecular assays	Triptonide activates the TNF signaling pathway.
[26]	Japan	70	Single-base extension multiplex assay	EGFR mutations in 13 cases (18.6%): RAS mutations in 10 (14.3%), EGFR in one (1.4%), and PIK3CA in 5 (7.1%). Concurrent gene mutations were found in three cases (4.3%). RAS mutations associated with shorter disease-free and overall survival; (*p* = 0.010) and (*p* = 0.024), respectively.
[27]	China	-	Functional annotation analysis	Identified 36 potential target miRNAs.
[28]	USA	20	RNA-sequencing	Frequency of translocations: t(6;9) > t(8;9) > t(8;14)
[29]	USA	-	RNA-seq	Three novel, truncated, domain-lacking FGFR1 variants cooperate with AXL and desensitize cells to FGFR1 inhibitor.
[30]	China, USA	55	Mouse model development, qRT-PCR, IHC	Deletion of both PTEN and SMAD4 may lead to ACC, mTOR activation and TGFβ1 overexpression (correlated with the aggressive solid ACC form)
[31]	USA	-	Single-cell RNA sequencing	Myoepithelial-to-ductal differentiation is promoted by RAR/RXR signaling.
[32]	Brazil	13	Rt-PCR	ACC cases exhibited elevated expressions of ITGB6, SMAD2, SMAD4, FBN1, LTBP1, and c-MYC.
[33]	USA	-	RNA-sequencing	ACC tumors utilize an alternative MYB promoter.
[34]	Germany	14	RNA-sequencing	Dysregulation of the Wnt signaling pathway and activation of the PI3K pathway were noticed in ACC.
[35]	Italy	4	DNA Intelligent Analysis	Forty-six miRNAs were differentially expressed between malignant and benign lesions.
[36]	Finland	38	IHC, rt-PCR, Genome-wide microarray	Two PP2A inhibitors were highly expressed in both dysplasia and malignancy, whereas positive p-S6 staining revealed activation of mTOR pathway.

## Data Availability

The original contributions presented in this study are included in the article. Further inquiries can be directed to the corresponding author.

## Abbreviation

Abbreviation	Acronym
MYB	v-myb avian myelobastosis viral oncogene homolog
NFIB	nuclear factor I/B
MYC	myelocytoma
NOTCH	neurogenic locus notch homolog protein
AXL	anexelekto
MET	mesenchymal epithelial transition factor receptor
EGFR	epidermal growth factor receptor
ATRA	all-trans retinoic acid
DEG	differential gene expression
RPPA	reverse-phase protein array
scRNA-seq	single-cell RNA sequencing
SPEN	solid pseudopapillary epithelial neoplasms
IHC	immunohistochemistry
PCR	polymerase chain reaction
HEY1	hes related family bHLH transcription factor with YRPW motif 1
EMT	epithelial-to-mesenchymal transition
MMP	matrix metalloproteinase
HES	hairy and enhancer of split
ceRNA	competing endogenous RNA
NGS	next-generation sequencing
PIK3CA	phosphatidylinositol-4,5-bisphosphate 3-kinase catalytic subunit alpha
ERBB2	erythroblastic leukemia viral oncogene homologue 2
HRAS	harvey rat sarcoma virus
TNF	tumor necrosis factor
ROS	proto-oncogene tyrosine-protein kinase
RAS	rat sarcoma
SOX	SRY-box transcription factor
AR	androgen receptor
SMAD	suppressor of mothers against decapentaplegic
MAPK	mitogen-activated protein kinase
RAD	radiation-sensitive protein
FGFR	fibroblast growth factor receptor
AKT	AKT serine/threonine kinase
FGF	fibroblast growth factor
PTEN	phosphatase and tensin homolog
TGF	transforming growth factor
mTOR	mechanistic target of rapamycin kinase
RAR	retinoic acid receptor
RXR	retinoid X receptor
ITGB	integrin subunit beta
FBN	fibrillin
LTBP	latent transforming growth factor beta binding protein
miRNA	micro ribonucleic acid
Wnt	wingless-type MMTV integration site
rt-PCR	reverse transcription polymerase chain reaction
PP2A	protein phosphatase 2 scaffold subunit Alpha
CIP2A	cellular inhibitor of protein phosphatase 2A (PP2A)
SET	SET nuclear proto-oncogene
EN1	engrailed homeobox 1
CTGF–new name CCN2	cellular communication network factor
SH-SY5Y	thrice-subcloned cell line derived from the SK-N-SH neuroblastoma cell line
IGF	insulin-like growth factor
SLC22A3	solute carrier family 22 member 3
FOXP2	forkhead box P2
Cdc42EP3	CDC42 effector protein 3
COL27A1	collagen type XXVII alpha 1 chain
DUSP1	dual specificity phosphatase 1
HSPB8	heat shock protein family B (small) member 8
Ki-67	marker of proliferation
hsa_circRNA	circular ribonucleic acid
FABP	fatty acid binding protein
VCAN	versican
KRAS	Kirsten rat sarcoma
BRAF	v-raf murine sarcoma viral oncogene homolog B
